# Local Diffusion Homogeneity (LDH): An Inter-Voxel Diffusion MRI Metric for Assessing Inter-Subject White Matter Variability

**DOI:** 10.1371/journal.pone.0066366

**Published:** 2013-06-11

**Authors:** Gaolang Gong

**Affiliations:** State Key Laboratory of Cognitive Neuroscience and Learning, Beijing Normal University, Beijing, China; Hangzhou Normal University, China

## Abstract

Many diffusion parameters and indices (e.g., fractional anisotropy [FA] and mean diffusivity [MD]) have been derived from diffusion magnetic resonance imaging (MRI) data. These parameters have been extensively applied as imaging markers for localizing white matter (WM) changes under various conditions (e.g., development, degeneration and disease). However, the vast majority of the existing parameters is derived from intra-voxel analyses and represents the diffusion properties solely within the voxel unit. Other types of parameters that characterize inter-voxel relationships have been largely overlooked. In the present study, we propose a novel inter-voxel metric referred to as the local diffusion homogeneity (LDH). This metric quantifies the local coherence of water molecule diffusion in a model-free manner. It can serve as an additional marker for evaluating the WM microstructural properties of the brain. To assess the distinguishing features between LDH and FA/MD, the metrics were systematically compared across space and subjects. As an example, both the LDH and FA/MD metrics were applied to measure age-related WM changes. The results indicate that LDH reveals unique inter-subject variability in specific WM regions (e.g., cerebral peduncle, internal capsule and splenium). Furthermore, there are regions in which measurements of age-related WM alterations with the LDH and FA/MD metrics yield discrepant results. These findings suggest that LDH and FA/MD have different sensitivities to specific WM microstructural properties. Taken together, the present study shows that LDH is complementary to the conventional diffusion-MRI markers and may provide additional insights into inter-subject WM variability. Further studies, however, are needed to uncover the neuronal mechanisms underlying the LDH.

## Introduction

Diffusion MRI is a unique, non-invasive method for exploring the anatomical and physical connectivity of the human brain through measuring the motion of water [1]. One key application of diffusion MRI is to infer the underlying fiber orientation within each voxel. The collective fiber orientations can then be used to reconstruct and extract white matter (WM) tracts. This process is known as diffusion tractography. The virtual WM tracts derived from diffusion tractography allow 3D fiber pathways to be rendered in an intuitive manner. This method has been increasingly used by neurosurgeons [2]. Moreover, diffusion tractography has made it possible to determine the presence and strength of the anatomical connections between grey matter (GM) regions and voxels *in-vivo*. These measurements are essential for reconstructing and analyzing human neuroanatomical networks [3], [4], [5]. A map of these neuroanatomical networks is the goal of the ongoing human connectome project [6].

Diffusion MRI also yields many diffusion parameters and indices that are putatively related to the microstructural properties of brain tissue (particularly WM) within the voxel. Fractional anisotropy (FA) and mean diffusivity (MD) are the most well known indices among these diffusion parameters, which are based on the diffusion tensor (DT) model [7], [8], [9]. Specifically, FA represents a normalized ratio of diffusion directionality, whereas MD quantifies the bulk mobility of water molecules. Other diffusion indices, such as radial diffusivity (RD), axial diffusivity (AD) and ellipsoidal area ratio (EAR), are also frequently applied [10], [11]. These diffusion indices are believed to reflect the biological fiber-packing density, fiber diameter, or the degree of myelination of the WM [12], [13]. Thus, these parameters are of great interest to neuroscience and clinical neurology. They have been extensively applied as markers for studying WM undernormal and pathological conditions [1], [14], [15].

The vast majority of diffusion MRI-derived indices (e.g., FA and MD), however, reflect diffusion properties solely within the voxel. Several studies have introduced inter-voxel diffusion indices that are informative and useful. For instance, the lattice index (LI) measures the similarities of the DTI-derived principle orientations between adjacent voxels [7]. This index has revealed significant region-specific WM changes under pathological conditions such as Alzheimer's disease [16], stroke [17], Williams syndrome [18], and multiple sclerosis [19]. In addition, a recent nonhuman primate study applied another inter-voxel index that measures the standard deviation of the angle of the first eigenvector projection within a neighborhood. This study demonstrated WM abnormalities during the chronic stage of induced cerebral ischemia [20]. Intriguingly, the studies that have applied both inter- and intra-voxel indices (e.g., FA and MD) have shown discrepant results between the indices. These results imply that these indices have different sensitivities to distinct WM properties. Furthermore, these discrepancies have been identified mainly under pathological conditions. The differences between the intra- and inter-voxel indices under normal conditions, however, have not yet been characterized. This analysis can provide important baseline information on the relationship between both types of indices.

Though very few inter-voxel metrics have been applied with diffusion MRI data to date, the metrics are uniformly based on diffusion tensor imaging (DTI) outputs. For instance, LI was defined as the degree of coherence of the principle diffusion directions derived from DTI [7]. However, it is well known that the DT model is problematic when assuming that a simple Gaussian profile can be applied to water diffusion in complex structures, such as in regions where fibers cross [21]. As a result, the existing DTI-derived inter-voxel indices (e.g., LI) are vulnerable to errors as a result of assuming false orientations, particularly in complex structural regions. Though non-DT models have been proposed to attempt to resolve the issues surrounding the analysis of complex structures [22], [23], [24], these models are mathematically complex and make quantifying model similarity (essential for defining an inter-voxel metric) difficult.

In the present study, we propose a novel inter-voxel metric referred to as local diffusion homogeneity (LDH), which is not dependent on any prior diffusion model. This metric is defined to capture the overall coherence of water molecule diffusion within a neighborhood, supposely reflecting the microstructural coherence of the underlying WM fibers. This proposed metric is expected to be complementary to the conventional diffusion parameters (e.g., FA and MD) and provide additional insights into the across-subject WM differences. To investigate its distinguishing features, the LDH metric will be systematically compared with the FA and MD metrics. Then, to illustrate potential applications, LDH and FA/MD will be both applied to measure age-related WM changes in a normal aging dataset. The results will be discussed.

## Methods

### Subjects

The present study included data from 23 young adults (males, 11; females, 12; age, 17–24 years) and 17 elderly individuals (males, 8; females, 9; age, 54–77 years). All subjects were recruited from the campus and the local community. Subjects with a history of neurological or psychiatric disorders were excluded from this study. The research protocol and consent procedure were approved by the Research Ethics Committee of the Beijing Normal University. Written informed consent was obtained from each participant.

### MRI acquisition

All scans were performed using the 3.0 T Siemens Tim Trio MRI scanner in the Imaging Center for Brain Research, Beijing Normal University. Diffusion MRI was acquired by using a single-shot echo planar imaging-based sequence. The diffusion MRI parameters included coverage of the whole brain; 2-mm slice thickness with no inter-slice gap; 68 axial slices; repetition time (TR), 9000 ms; echo time (TE), 92 ms; 64 optimal nonlinear diffusion-weighted directions with b = 1000 s/mm^2^ and additional images without diffusion weighting (i.e., b = 0 s/mm^2^); number of average, 4; acquisition matrix, 128×124; field of view (FOV), 256×248 mm^2^.

### Image pre-processing

To correct for eddy-current induced image distortion and simple head motion, the diffusion-weighted images (DWI) were first coregistered to a reference volume (i.e., the b0 image) using an affine transformation. The voxel-wise diffusion tensor matrix was then calculated for each subject in the native space. Next, diagonalization was performed to yield three pairs of eigenvalues and eigenvectors. Based on the three eigenvalues, fractional anisotropy (FA) and mean diffusivity (MD) were computed on a voxel-by-voxel basis. To compare between subjects, the framework of Tract-Based Spatial Statistics (TBSS) was used to establish the WM correspondence between subjects [25]. Specifically, the FA image of each subject was nonlinearly registered to the FMRIB58_FA template in the standard space. The mean of all aligned FA images was then calculated, and the WM skeleton was generated based on the mean FA image. As recommended, the WM skeleton was thresholded at 0.2. This step led to a binary skeleton mask. All FA and MD data were then projected onto the skeleton mask. The TBSS framework avoids the necessity of choosing a spatial smoothing procedure, a step that is required for typical voxel-based analysis (VBA). In addition, the framework provides better alignment, sensitivity, objectivity and interpretability when it is applied to multi-subject diffusion data [25]. For these reasons, our analyses of FA/MD/LDH were confined to the WM skeleton mask derived from the TBSS. All of the procedures described above were implemented by the PANDA toolbox [26].

### Local diffusion homogeneity (LDH)

For a diffusion MRI dataset, the diffusivity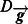 along the diffusion-weighted gradient direction 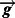 can be calculated as follows: 
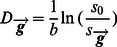
(1)


**Figure 1:**
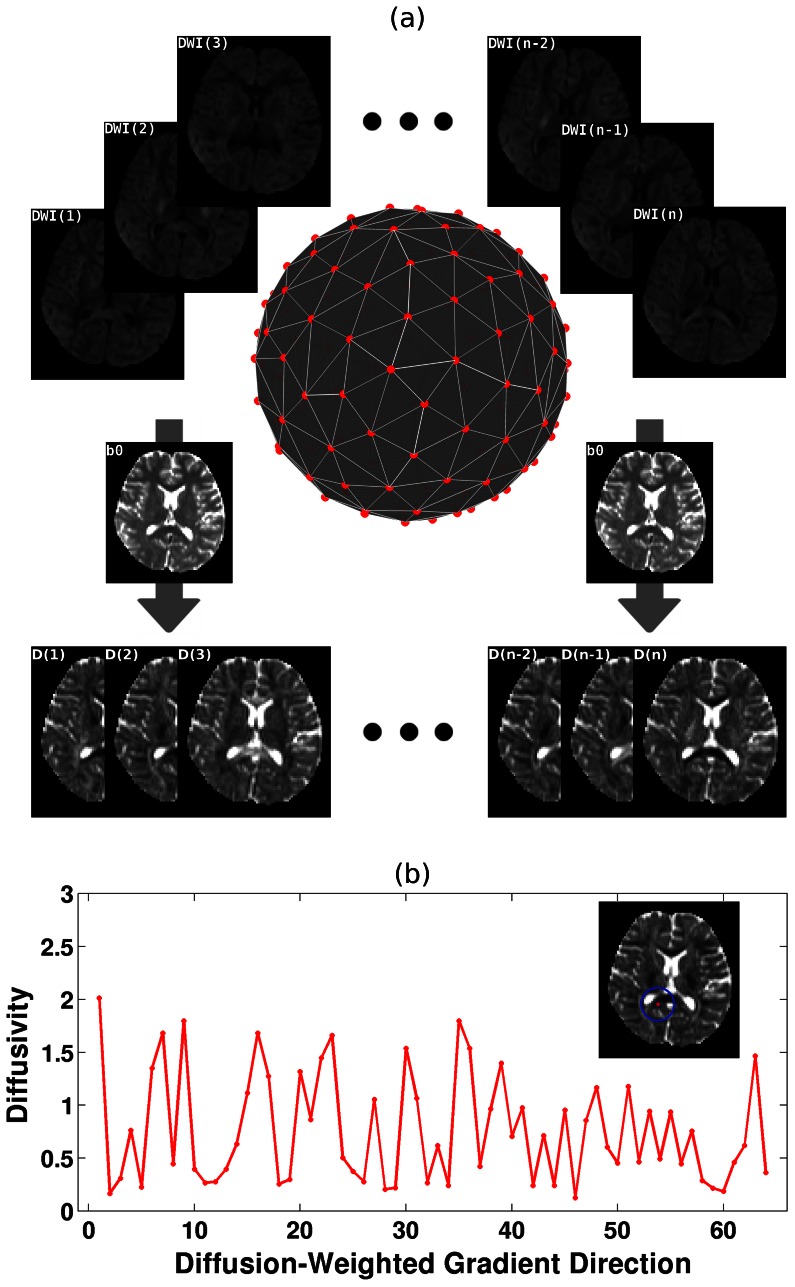
The extraction of voxel-wise diffusivity series. (a) Schematic diagram showing the processing procedures for calculating the diffusivity (D) along the gradient directions. The vector from the center of the sphere to a red circle on the surface represents a diffusion gradient direction. For each gradient direction, one diffusion-weighted image (DWI) was collected and used to calculate the diffusivity images. (b) The diffusivity series for a sample voxel (the red voxel). A dataset with 64 gradient directions was analyzed.

Where 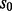 is the signal without diffusion weighting (i.e., b = 0 s/mm^2^); 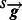 is the signal along the gradient direction 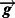 with diffusion weighting and ***b*** is the weighting factor (i.e., b-value in the sequence). For each gradient direction, the voxel-wise 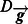 was computed after correcting for the eddy-current artifacts in the original DWI. Given that multiple gradient directions are sampled in each diffusion MRI scan, a series of 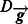 can be extracted for each voxel (Fig 1). The length of the series (i.e., the sample size) is simply the total number of sampled gradient directions.

The LDH of a given voxel was defined as the overall similarity of the 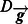 series within its nearest neighborhood. The neighborhood can be 7 voxels (including the neighbors adjacent to the surface), 19 voxels (including the neighbors adjacent to the surface and edge) or 27 voxels (including the neighbors adjacent to the surface, edge and vertex) in the 3D image space. Kendall’s coefficient concordance (KCC) [27] was applied to quantify this overall similarity (i.e., LDH) as follows: 
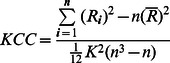
(2)

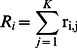



**Figure 2:**
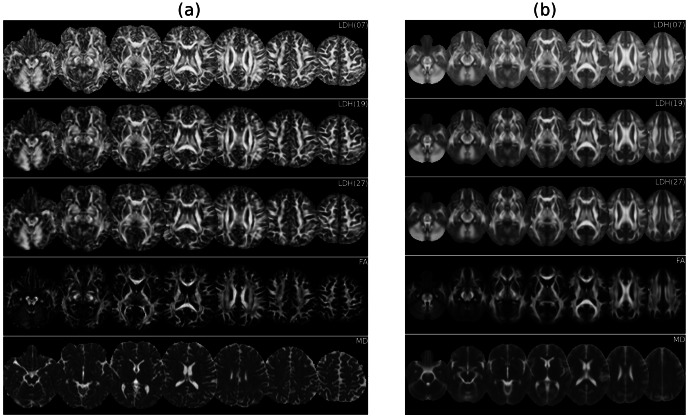
LDH and FA/MD across the entire brain. (a) Representative LDH and FA/MD images from one subject in the native space. The number in the bracket (i.e., 7, 19, 27) represents the size of the nearest neighborhood. (b) The average LDH and FA/MD images across the entire sample of 40 subjects. The images were nonlinearly registered into the standard space using the TBSS framework.

Where 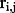 represents the rank of the 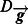 of ***i***-th diffusion gradient direction in the entire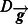 series for the ***j***-th voxel in a defined neighborhood; 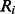 is the sum of the 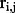 for all K voxels within the neighborhood and 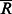 is the mean of 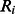 for all n diffusion gradient directions; K is the number of voxels for the pre-defined neighborhood (i.e., 7, 19 or 27); n is the length of the 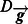 series (e.g., 64 in the current dataset). The KCC is a non-parametric statistic ranging from 0 (no agreement) to 1 (complete agreement), here representing the overall similarity of the 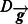 series within the neighborhood. The LDH maps with the three types of neighborhoods (i.e., 7, 19 or 27) were calculated for each subject in the native space (Fig 2). To compare between subjects, the LDH images were then projected onto the WM skeleton mask in the standard space using the procedure for the TBSS framework described above.

To test the effect of the gradient direction number (i.e., sample size) on the reliability of LDH, diffusion-weighted gradient directions from a total of 64 gradient directions were sampled from the dataset. The sample size was defined as a number from 6 to 63 because 6 represents the minimum number of gradient directions in any DTI acquisition. For each sample size, random sampling was repeated 50 times for each of the 40 subjects. For each sample, the LDH images were then calculated using the procedures described above. To quantify the reliability of LDH for each sample size, the intra-class coefficient (ICC) was computed, as shown below [28]:
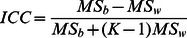
(3)


**Figure 3:**
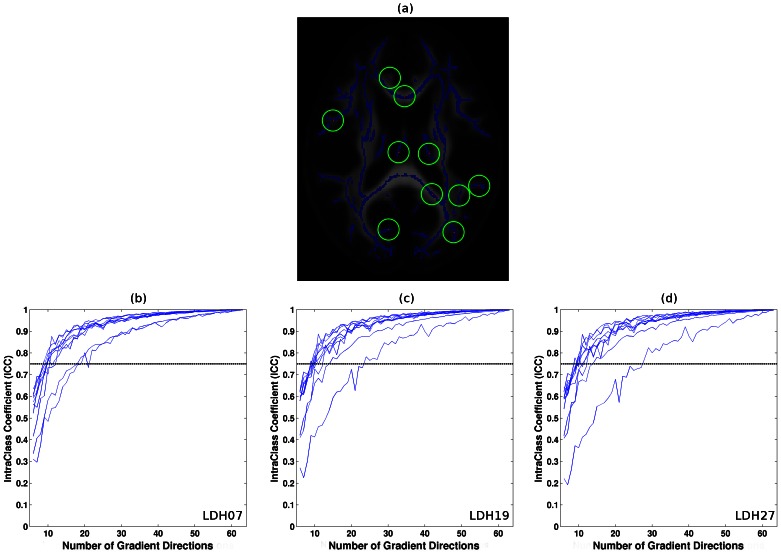
The effect of diffusion-weighted gradient direction number on the reliability of LDH. (a) Ten voxels (red) were randomly selected to test the reliability of LDH. (b)–(d) Intra-class coefficient (ICC) of LDH07, LDH19 and LDH27 as a function of the number of diffusion-weighted gradient directions (6∼63). The curves represent the mean ICC minus 1std (i.e., mean-1std) of ICC across the samples. This result represents the lower limit (i.e., the worst case) of ICC across samples (50 in total). Each curve is calculated for one voxel (10 in total). The cutoff value for the criterion of excellent reliability (i.e., ICC = 0.75) is denoted by the grey dashed line.

Where 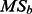 is the between-subject variance of the LDH for a given sample size, 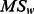 is the within-subject variance and ***K*** is the number of repeated observations per subject. Notably, the LDH value measured from the entire set of gradient directions (i.e., the reference) and the LDH value measured from each sample were considered two repeated observations (i.e., ***K*** = 2). Therefore, each sample with a given sample size had a calculated ICC value for each voxel (50 in total). To avoid the intensive computation and disk-space requirements of the resampling procedures (40×50×58×3 images), 10 voxels were randomly selected on a random slice in the standard space (Fig 3). These voxels were then inversely transformed back to the native space of each subject. Using the corresponding data in the native space, the ICC values were then calculated for the 10 voxels. An ICC value ranging from 0.75 to 1 is considered to reflect excellent reliability [29], [30].

### Correlations between LDH and FA/MD

To evaluate the similarity between the across-space variabilities of the LDH and FA/MD metrics, Pearson correlations were computed to compare across the voxels on the WM skeleton mask (123081 voxels in total) for each subject. For each voxel on the WM skeleton mask, Pearson correlations were also computed to compare between different subjects (40 in total), indicating the similarity between the across-subject variabilities of the LDH and FA/MD metrics. The False Discovery Rate (FDR) [31] was applied to correct for multiple comparisons and a two-tailed value of p<0.05 after correction was considered to be significant.

### Group comparison between young adults and elderly individuals

To demonstrate the usability of LDH in studying between-group WM differences, the LDH values of each voxel on the WM skeleton mask were statistically compared between the young adults (23 subjects) and elderly individuals (17 subjects) using a general linear model (GLM). Gender was controlled as a covariate in the statistical model. The same GLM was also applied to the FA/MD analysis. Given the well-known declines of WM integrity in elderly, a one-tailed value of p<0.05 after FDR correction was considered to be statistically significant. The statistical results between the LDH and FA/MD metrics were further compared.

## Results

Sample LDH, FA and MD maps are illustrated in Fig 2. The LDH maps visually resemble the FA images and display contrasts between WM, GM and CSF. However, the degree of tissue contrast in the LDH map is different from that observed in the FA map. In contrast, the LDH images appear quite different than the MD images.

### The effect of gradient direction number

Next, the effect of diffusion-weighted gradient direction number on the reliability of LDH was quantified. The ICC was calculated for 10 randomly selected voxels (Fig 3). The LDH of the entire set of diffusion gradient directions was used as a reference, and an ICC value (50 in total, each for one sample) was calculated for each sample with a given sample size (i.e., 6∼63). Next, the mean ICC minus the ICC standard deviation (i.e., mean-1std) was plotted as a function of sample size for each voxel, indicating the lower limit (i.e., the worst case) of LDH reliability across the samples. As shown in Figure 3, the LDH ICC curves exhibited an overall increasing trend that corresponded with an increase in sample size. The LDH reliability for all 10 voxels reached the level of excellence (i.e., ICC>0.75) when the number of gradient directions exceeded 20∼30.

### Correlations between the LDH and FA/MD metrics across space

**Figure 4:**
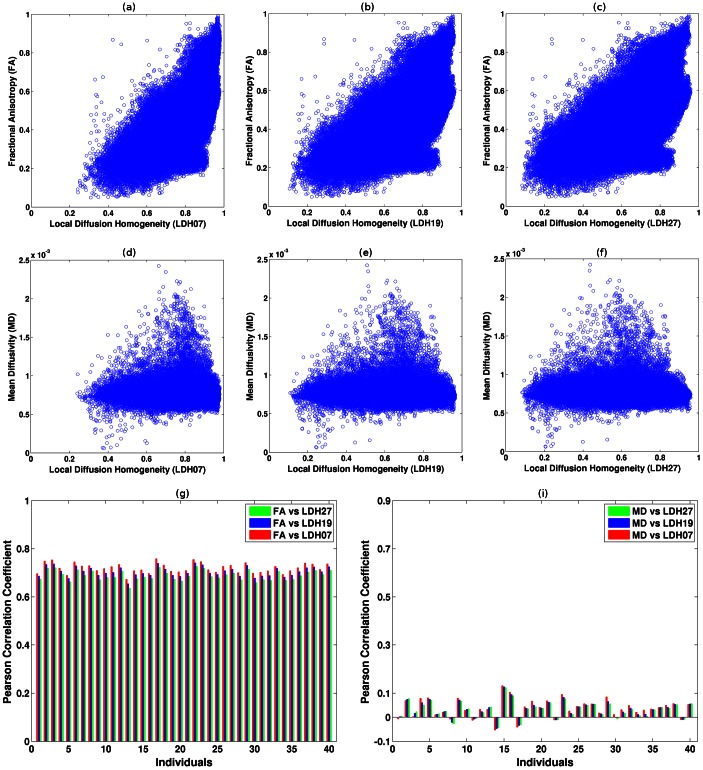
Correlations between the LDH and FA/MD metrics across space. (a)–(f) Representative scatter plot between the LDH and FA/MD metrics across the WM skeleton of one subject. Each circle represents one voxel on the skeleton. (g)–(i) The Pearson correlation between the LDHs and FA/MD metrics for all subjects.

A scatter plot comparing LDH and FA/MD across the WM skeleton is depicted in Figure 4a–f. For each subject, the Pearson correlation coefficient *R* was calculated (LDH07 vs. FA: mean = 0.72, std = 0.02; LDH19 vs. FA: mean = 0.70, std = 0.02; LDH27vs. FA: mean = 0.69, std = 0.02; LDH07 vs. MD: mean = 0.04, std = 0.04; LDH19 vs. MD: mean = 0.03, std = 0.04; LDH27 vs. MD: mean = 0.03, std = 0.03). The results of this analysis are displayed in Figure 4 g–i. The results indicate that the LDH and FA metrics exhibit similar across-space contrasts to some degree within the WM skeleton mask. However, the LDH and MD metrics did not exhibit similar across-space contrasts.

### Correlations between LDH and FA/MD across subjects

**Figure 5:**
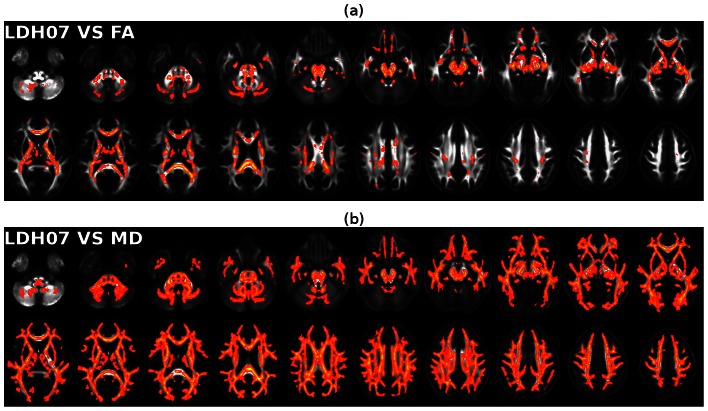
Correlations between the LDH07 and FA/MD metrics across subjects. To highlight the potential differences between the metrics, voxels that did not exhibit significant correlations (p>0.05, FDR corrected) between the metrics were color-coded. (a)–(b)The panels depict the spatial pattern of voxels exhibiting non-significant correlations between LDH07 and FA as well as LDH07 and MD. The statistical analysis was performed on the WM skeleton derived from TBSS. The results were ‘thickened’ for better visualization (http://www.fmrib.ox.ac.uk/fsl/tbss/index.html#display).

As listed in Table 1, 79.62%, 75.86% and 72.93% of the voxels on the WM skeleton showed significant linear correlations (p<0.05, FDR corrected) between LDH07, LDH19 and LDH27 and FA, respectively. To highlight the differences in the spatial patterns revealed by the LDH07 and FA metrics, the voxels showing p>0.05 after FDR correction (i.e., non-significant) are displayed in Figure 5a. The voxels that were not significantly correlated between the LDH07 and FA metrics were mainly located in the cerebellar WM, cerebellar peduncle, corticospinal tract, internal capsule, thalamic radiation, and corpus callosum.

**Table 1:** The percentage of voxels showing significant correlations (p<0.05, FDR corrected) between the LDH and FA/MD metrics on the WM skeleton (123081 voxels in total).

	FA	MD
LDH07	79.62%	3.77%
LDH19	75.86%	2.84%
LDH27	72.93%	2.44%

In contrast, only 3.77%, 2.84% and 2.44% of the voxels showed significant correlations between LDH07, LDH19 and LDH27 and MD, respectively. The voxels that did not exhibit significant correlation between the LDH07 and MD metrics are illustrated in Figure 5b. These areas included most of the WM regions. The spatial patterns revealed by correlations between LDH19/LDH27 and FA/MD are extremely similar to those of LDH07 and are not shown.

### Applying the LDH metric to examine normal aging: between-group WM differences between young adult and elderly individuals

**Figure 6:**
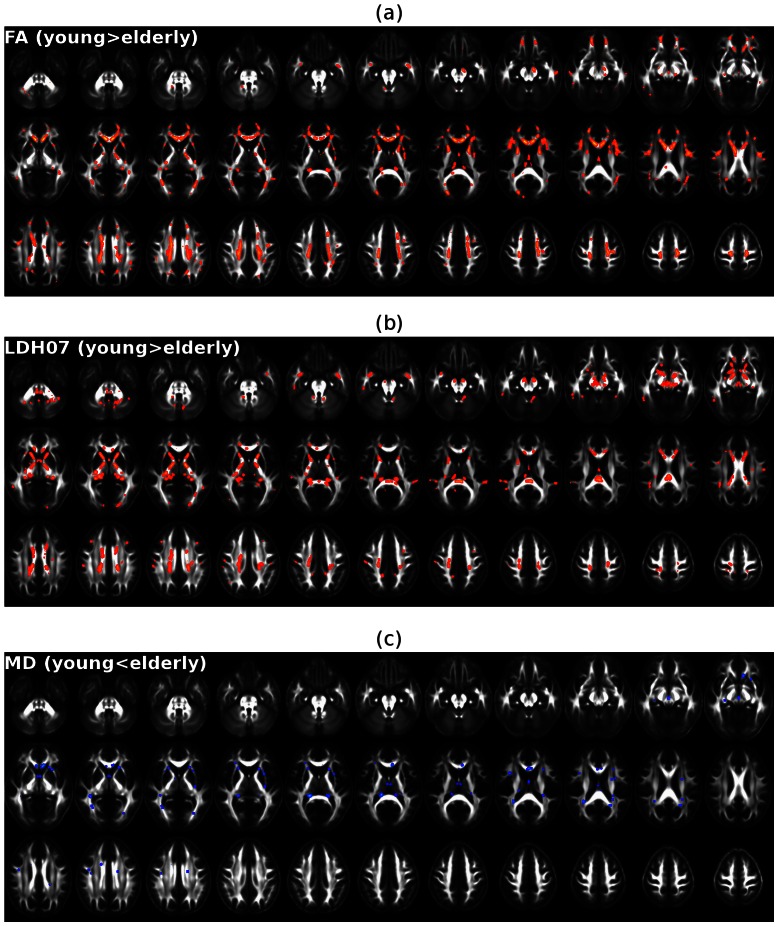
The significant age-related WM changes revealed by the LDH, FA and MD metrics. (a) Significant FA reduction (p<0.05, FDR corrected) in elderly individuals. (b) Significant LDH07 reduction (p<0.05, FDR corrected) in elderly individuals. (c) Significant MD increase (p<0.05, FDR corrected) in elderly individuals. The statistical analysis shown in this figure was performed only on the WM skeleton derived from TBSS. The results were ‘thickened’ for better visualization.

Statistical maps showing significant group differences (p<0.05, FDR corrected) in FA, MD and LDH07 are illustrated in Figure 6. Specifically, elderly individuals exhibited significantly decreased FA in multiple regions throughout the brain. FA was mainly affected in the bilateral uncinate fasciculus, superior longitudinal fasciculus, internal capsules, external capsules, fornices and corpus callosum (Fig 6a). The regions with reduced LDH07 in the elderly individuals were mainly located in the bilateral cerebellar WM, uncinate fasciculus, cerebral peduncles, internal capsules, fornices, superior longitudinal fasciculus, and corpus callosum (Fig 6b). In contrast, MD was significantly increased in the elderly individuals. The bilateral external capsules, fornices, superior longitudinal fasciculus, and corpus callosum (Fig 6c) were preferentially affected.

**Figure 7:**
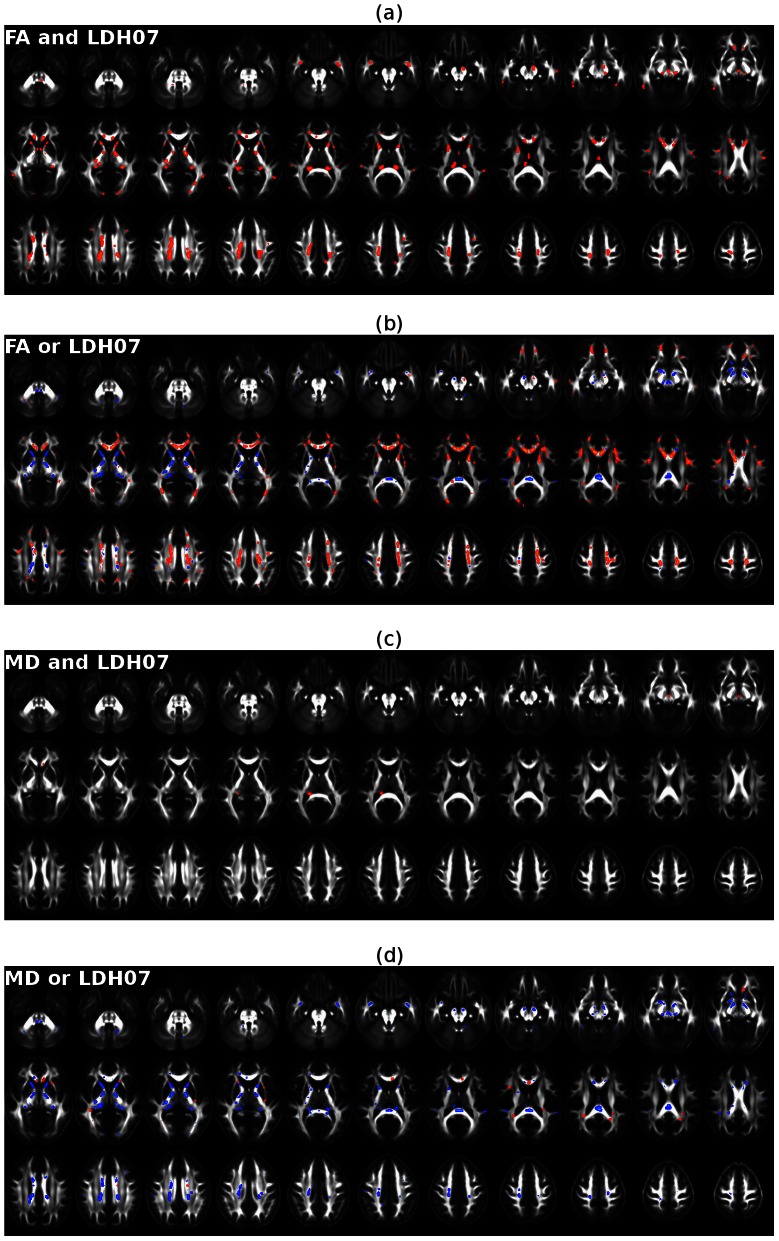
Maps showing the spatial consistencies and inconsistencies between LDH and FA/MD in the detection of age-related effects. (a) The voxels showing significant age-related effects that were detected by both FA and LDH07. (b) The voxels showing significant age-related effects that were detected by either FA or LDH07 alone. The yellow-red represents significant age-related effects that were only detected by FA but not on LDH07. Bright blue represents significant age-related effects that were only detected by LDH07 but not FA. (c) The voxels showing significant age-related effects that were detected by both MD and LDH07. (d) The voxels in which an age-related effect was only detected by either MD or LDH07. The yellow-red represents significant age-related effects that were detected only by MD but not by LDH07. Bright blue represents significant age-related effects that were only detected by LDH07 but not MD. All of these results were also ‘thickened’ for visualization.

Though the WM regions showing significant changes in LDH07 and FA/MD overlapped to some degree, the exact location of the significant changes greatly differed (Fig 6). As summarized in Table 2, only 34.3%, 35.3% and 35.3% of the voxels with significant reductions in LDH07, LDH19 and LDH27 also showed significant FA reductions, respectively. Furthermore, less than 2% of the voxels showing significant LDH changes intersected significant MD changes (Table 2). Maps showing the consistent and inconsistent results between the LDH07 and FA/MD metrics are illustrated in Figure 7. Specifically, the age-related WM voxels that differed in LDH07, but not in FA/MD, were mainly located in the bilateral cerebellar peduncles, internal capsules, fornices (inferior part), and splenium of the corpus callosum (Fig. 7b and 7d).

**Table 2:** Voxels showing significant age-related effects as measured by both the LDH and FA/MD metrics (FDR corrected p<0.05).

	FA	MD
	(Elderly>young, 6965 voxels)	(Elderly<young,180 voxels)
**LDH07** (Elderly>young, 4711 voxels)	Intersection: 1615 voxels	Intersection: 66 voxels
	Percentage: 34.3% LDH07	Percentage: 1.4% LDH07
	Percentage: 23.2% FA	Percentage: 36.7% MD
**LDH19** (Elderly>young, 3910 voxels)	Intersection: 1379 voxels	Intersection: 64 voxels
	Percentage: 35.3% LDH19	Percentage: 1.6% LDH19
	Percentage: 19.8% FA	Percentage: 35.6% MD
**LDH27** (Elderly>young, 3697 voxels)	Intersection: 1304 voxels	Intersection: 62 voxels
	Percentage: 35.3% LDH27	Percentage: 1.7% LDH27
	Percentage: 18.7% FA	Percentage: 34.4% MD

## Discussion

Using diffusion MRI, the present study has proposed a novel inter-voxel metric referred to as local diffusion homogeneity (LDH). This measure can be applied as an imaging marker for characterizing the WM microstructural properties of the human brain in a non-invasive manner. The comparative analysis between LDH and FA/MD showed that LDH resembles an index of diffusion anisotropy. However, it revealed unique inter-subject variability in specific WM regions. When applying these indices to the detection of age-related WM alterations, the significant differences depicted by LDH and FA/MD in multiple WM regions were not consistently detected. This result suggests that each of these indices is differentially sensitive to specific WM microstructural properties.

### Definition of LDH

To date, few diffusion inter-voxel metrics have been proposed. One such metric is lattice index (LI), which mainly characterizes the overall coherence of principle orientations derived from diffusion tensors (DT) [7]. These indices, however, may erroneously estimate coherence when the DT model fails to provide the real tract direction within the voxel. These errors are especially pertinent in complex structures such as regions of fiber-crossing. In contrast, the proposed LDH metric does not require a prior diffusion model. Instead, the model-related issues are avoided through the quantification of the raw diffusivity homogeneity along the sampled gradient directions. In addition, by using the signals from the entire set of gradient directions, the LDH metric actually measures the inter-voxel similarity of the full diffusion profile of water molecules. In contrast, previous metrics like LI measure the inter-voxel similarity of the principle diffusion orientations.

Since the full profile of water molecule diffusion is highly related to underlying WM microstructural properties (fiber direction, fiber coherence, fiber density, fiber diameter, degree of myelination, degree of fiber crossing, etc.), the LDH metric is supposed to reflect specific microstructural properties of the underlying WM fibers, by capturing the overall coherence of water molecule diffusion within a neighborhood. For instance, within a specific WM tract, the fiber orientations around the curving part are locally incoherent, thus leading to a less LDH value on the curving part than the straight part. In this case, the LDH metric provides useful clues about the coherence of local fiber orientations, as well as local shape of the WM tract. On the other hand, the fiber meylination, diameter or density differs along each WM tract [32], it is intuitive to assume that the LDH metric can represent the local coherence of those microstructural factors within the neighborhood, another meaningful information for understanding the microstructure of WM tracts. Notalby, these biological interpretations for the LDH metric are largely speculative. To validate these, future studies with histological analyses are required.

Computationally, the LDH metric uses Kendall's coefficient concordance (KCC) to quantify the overall coherence of the diffusivity series. The KCC is a non-parametric statistic that can be calculated without assumptions about the nature of the probability distribution. It is linearly related to the mean value of the Spearman's rank correlation coefficients between all pairs of the rank series within a neighborhood. Thus, the KCC is an ideal candidate for measuring the coherence of the diffusivities that are frequently distributed in a non-Gaussian pattern, which is the case for complex structures. It is worth mentioning that the usage of KCC in the present study was directly inspired by a previous fMRI study by Zang et al. [33] in which the KCC was employed to measure the similarities between fMRI time series in grey matter (GM). Follow-up studies have further demonstrated that the KCC of fMRI time series are altered in many neurological diseases, including Alzheimer's disease [34], attention deficit hyperactivity disorder [35] and Parkinson disease [36]. These findings suggest that there is a region-specific distribution of abnormal neuronal activity in these diseases.

Furthermore, it should be noted that two factors can affect LDH results: 1) the choice of the neighborhood and 2) the number of gradient directions. In the present study, three neighborhoods (i.e., 7, 19 and 27) were tested in the analyses. Not surprisingly, differences between the LDH of the three types of neighborhood were identified. For example, the LDH of the voxels in the neighborhood of 7 exhibited the highest correlations with FA across space (Fig 3). The LDH detected in this neighborhood were also most consistent with the FA-detected WM changes in normal aging (Table 2). Despite these slight differences, the overall patterns of LDH were quite similar between the three neighborhoods. This result implies that the choice of neighborhood only has a limited effect on the results.

In contrast, the number of gradient directions measured is critical in the determination of LDH. This variable can substantially affect the reliability of LDH. Currently, the number of gradient directions that is adequate for the reliable detection of WM properties by diffusion MRI is debated. Many studies have been dedicated to answering this question and have measured the reliability and robustness of DTI-derived parameters (e.g., FA, MD, etc.) [37], [38]. In the present study, the sampling results suggested that 20∼30 gradient directions was adequate for achieving high LDH reliability (Fig 3). A specific cut-off, however, is difficult to determine and unnecessary. Many current studies commonly apply 20∼30 gradient directions for their diffusion MRI acquisitions. Thus, the LDH metric is highly applicable to existing diffusion MRI datasets. Moreover, diffusion spectrum imaging [39] and Q-ball imaging [40] are promising techniques for resolving the issues involving complex structures. These techniques have been increasingly applied in the recent years and provide diffusion MRI datasets that naturally fit well with the LDH analysis.

### LDH versus FA/MD

In this study, we performed a systematic comparison between the LDH and FA/MD metrics in a normal sample. The information that we have gleaned from our experiments can provide important baseline information about the relationships between these indices. The results indicate that there exists some degree of overlap between the LDH and FA metrics. In contrast, LDH did not correlate with MD. It is worth noting that LDH should not be viewed as a real ‘anisotropy’ index because it actually measures the inter-voxel coherence of water molecule diffusion profiles. In fact, there are appreciable differences between LDH and FA across space and normal subjects (Fig 4 and Fig 5). Furthermore, the FA and LDH metrics each identified discrepant WM brain areas as being affected by aging.

The observed between-index differences are consistent with previous findings. Other studies have reported notable discrepancies between other inter-voxel parameters and the FA/MD metrics under pathological conditions. For instance, the lattice index (LI) revealed a greater difference between tumor models and between tumor and peritumoral regions than did the FA/MD measurements. This characteristic of LI can be useful for differentiating between tumor models and between tumors from and peritumoral regions [41]. In addition, the inter-voxel metric measuring *the standard deviation of the angle of the first eigenvector projection within a neighborhood* revealed WM abnormalities in the chronic phases of ischemia. In this study, the FA/MD metrics failed to uncover any WM differences [20]. Together, these reports and the current study suggest that the inter-voxel indices (e.g., LDH) may be applied when no significant results are found by assessing FA/MD.

Multiple microstructural factors may contribute to various diffusion metrics in WM. These factors include the fiber density, fiber diameter, fiber coherence and degree of myelination [10], [13]. It is conceivable that the diffusion parameters are each sensitive to a specific subset of the factors. In other words, each parameter may preferentially reflect some of the factors over the others. Speculatively, LDH may be more sensitive to the microstructural coherence but less sensitive to the degree of myelination than FA. Given the differential sensitivities of the different metrics, LDH may reveal WM microstructural changes that FA/MD fails to detect and vice versa. Therefore, LDH and FA/MD should be applied as complementary approaches when exploring inter-subject WM variability.

### WM changes during normal aging

To demonstrate the utility of LDH in an experimental setting, LDH and FA/MD were measured to reveal the WM alterations present in normal elderly individuals. Interestingly, significant FA reductions were found in the bilateral uncinate fasciculus, superior longitudinal fasciculus, external capsules, fornices and corpus callosum. Our results are highly compatible with previous findings [42], [43]. In contrast, MD also displayed significant changes, but in fewer WM voxels. This result supports the notion that diffusion metrics are distinctly sensitive to specific WM properties. Notably, age-related FA reductions are primarily distributed in the anterior part of the WM. This result is consistent with the hypothesis of the aging-related anteroposterior gradient. This hypothesis states that the anterior regions of brain are more susceptible to aging than the posterior regions [43]. These FA/MD changes are most likely a reflection of age-related axonal shrinkage, axonal loss or demyelination. These processes may underlie various patterns of cognitive decline during normal aging.

In addition to the FA/MD changes, LDH was also significantly reduced throughout the brains of the elderly individuals. As expected, whereas some WM voxels exhibited age-related changes that were detected by both the LDH and FA/MD metrics, others were only detected by LDH. The age-related WM alterations detected only by LDH were mainly located in the bilateral cerebellar peduncles, cerebral peduncles internal capsules, fornices (inferior part), and splenium of the corpus callosum. These data provide new avenues for understanding the aging brain. The changes in these WM regions during aging are most likely a result of the disruption of fiber coherence, alteration of the tract shape, or the reduction of the tract size. They are less likely a result of axonal shrinkage or demyelination. In fact, several studies have reported morphological WM changes in these regions. For example, the volumes of the cerebellar peduncle and splenium are decreased in the aging brain [44]. Further studies will need to be performed to fully interpret these findings and assess the reliability of the current age-related LDH results.

### Limitations

Several limitations in the present study should be addressed. First, while the proposed LDH metric showed a potential for assessing WM differences across individuals, the biological interpretations are largely speculative and specific neuronal basis for this new metric remains unknown. To resolve this, virtual validation analyses with histological data or phantoms are necessary, which is however beyond the scope of the present study. Such validation studies are highly desired in the future. Second, the current LDH analysis was restricted to the WM skeleton constructed using the TBSS framework. Thus, the WM regions off of the skeleton and GM regions are excluded. However, the TBSS framework was employed because of its high quality across-subject registration procedure. This process minimizes alignment issues when comparing between young adults and elderly individuals. The skeletonizing technique also minimizes partial volume effects. Third, we only applied the LDH metric to study age-related WM changes. More datasets, particularly those from individuals with brain disorders (e.g., schizophrenia, multiple sclerosis and et al.), are needed to further demonstrate the utility of LDH. Finally, the subject sample size was relatively small in the present study. Future studies with larger sample size are required to test the reproducibility of our current findings.

## Conclusion

In the present report, we have proposed a novel diffusion MRI-derived metric referred to as local diffusion homogeneity (LDH). This metric is defined to characterize the overall coherence of water molecule diffusion within a neighborhood, and can be used to explore inter-subject variability of WM microstructural properties. In contrast with the more commonly applied metrics, such as FA and MD, LDH is a model-free inter-voxel index, and may reveal WM microstructural alterations that FA/MD fails to detect. Thus, it is complementary to the conventional diffusion-MRI markers and may provide additional insights when applied to study inter-subject WM variability.
